# Consideration of COVID-19 beyond the human-centred approach of prevention and control: the ONE-HEALTH perspective

**DOI:** 10.1080/22221751.2022.2125343

**Published:** 2022-10-26

**Authors:** Qin Li, Robert Bergquist, Liz Grant, Jun-Xia Song, Xin-Yu Feng, Xiao-Nong Zhou

**Affiliations:** a School of Global Health, Chinese Center for Tropical Diseases Research, Shanghai Jiao Tong University School of Medicine; One Health Center, Shanghai Jiao Tong University-The University of Edinburgh, Shanghai, People’s Republic of China; b Chinese Center for Disease Control and Prevention (Chinese Center for Tropical Diseases Research); NHC Key Laboratory of Parasite and Vector Biology; WHO Collaborating Centre for Tropical Diseases; National Center for International Research on Tropical Diseases, National Institute of Parasitic Diseases, Shanghai, People’s Republic of China; c Ingerod, Brastad, Sweden (formerly at the UNICEF/UNDP/World Bank/WHO Special Programme for Research and Training in Tropical Diseases (TDR), World Health Organization, Geneva, Switzerland; d Global Health, The University of Edinburgh, Edinburgh, UK; e Food and Agriculture Organization of United Nations, Rome, Italy; f Department of Biology, College of Life Sciences, Inner Mongolia University, Hohhot, People’s Republic of China

**Keywords:** COVID-19, human, animal, One health

## Abstract

Most of the new emerging and re-emerging zoonotic virus outbreaks in recent years stem from close interaction with dead or alive infected animals. Since late 2019, the coronavirus disease 2019 (COVID-19) has spread into 221 countries and territories resulting in close to 300 million known infections and 5.4 million deaths in addition to a huge impact on both public health and the world economy. This paper reviews the COVID-19 prevalence in animals, raise concerns about animal welfare and discusses the role of environment in the transmission of COVID-19. Attention is drawn to the One Health concept as it emphasizes the environment in connection with the risk of transmission and establishment of diseases shared between animals and humans. Considering the importance of One Health for an effective response to the dissemination of infections of pandemic character, some unsettled issues with respect to COVID-19 are highlighted.

## Background

The United Nations Environment Programme (UNEP) stated in 2016 that the main source of emerging and re-emerging zoonotic diseases may have a correlation with deforestation, intensive farming, illegal and poorly regulated wildlife trade, and climate change [1]. Indeed, as many as 75% of new infectious diseases originate as a direct result of human-animal interaction [2], and Zika, Ebola, epidemic nephropathy, avian influenza, severe acute respiratory syndrome coronavirus (SARS), Middle East respiratory syndrome (MERS) have all come from various animal reservoirs. The second coronavirus (SARS-CoV-2), the cause of the current pandemic coronavirus disease 2019 (COVID-19), is no exception. As of 1 January 2022, COVID-19 has caused more than 290 million cases and close to 5.5 million deaths [3]. Despite preventive measures, such as general lockdowns and personal measures, e.g. disinfection, face cover, and isolation together with medication and large-scale vaccination, the COVID-19 situation in the world has rather worsened than improved.

**Figure 1.**
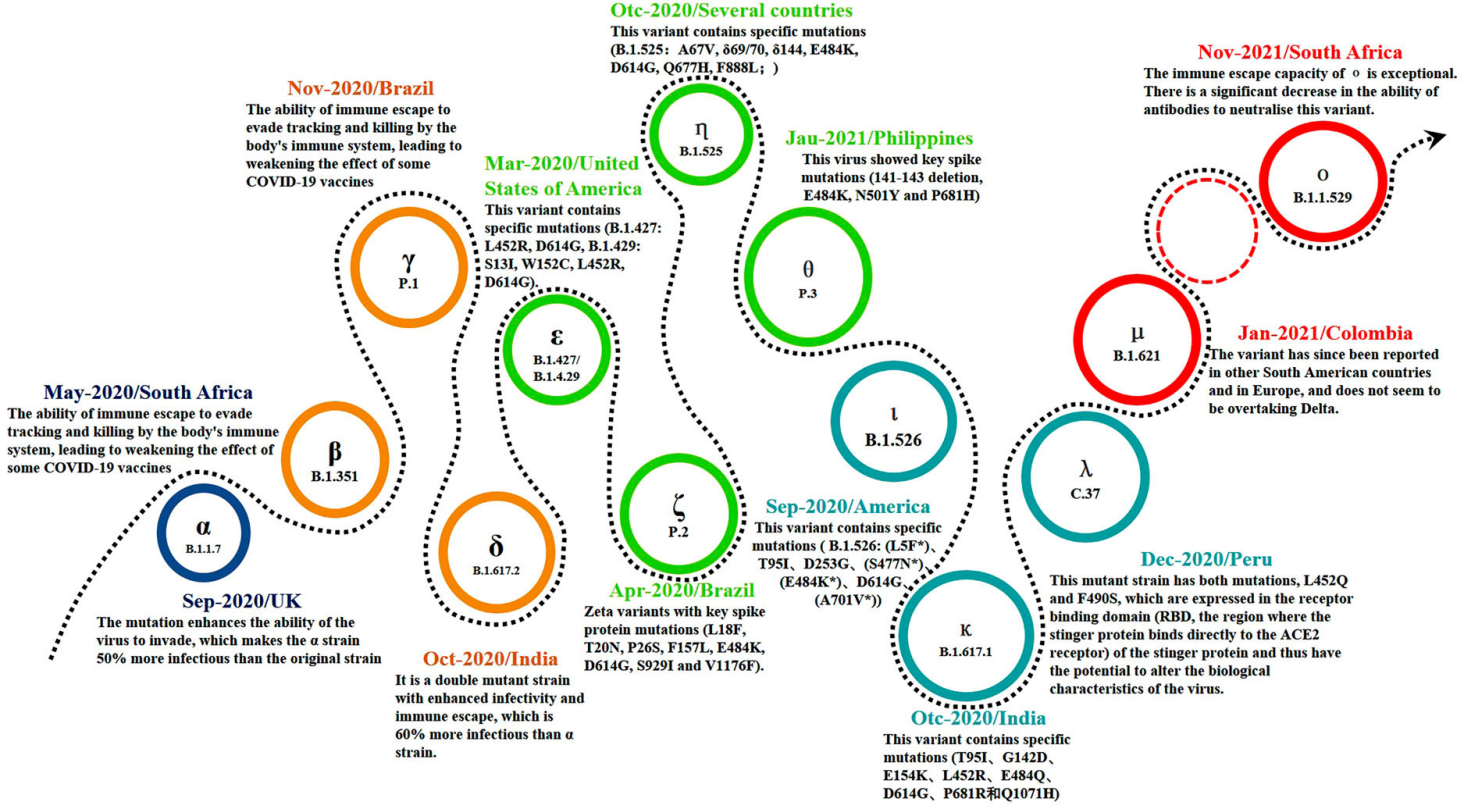
Major, novel SARS.CoV-2 variants worldwide.

Since its first manifestation as an unusual from of pneumonia in Wuhan, China in late 2019 [4], COVID-19 has appeared in waves supported by sustained, rapid transmission throughout the world. The causative virus, first isolated in January 2020 [5], has been shown to be similar to another severe, acute, respiratory syndrome coronavirus (SARS) that caused an epidemic in China and Southeast Asia about 20 years ago [6]. Following the disclosure of the whole-genome sequence of SARS-CoV-2 in early 2020 [7], massive mutations had already started to occur and there are now an untold number of mutant strains, with 13 major ones (including the latest Omicron variant). New variants continue to emerge (Figure 1) and governments worldwide, aware of the extreme risks posed, have taken strong measures to deal with the challenges of the ongoing confrontation with the SARS-CoV-2 virus.

Current control policies (mandated or recommended) are primarily based on a human-centred framework focused on identification of new cases leading to surveillance and rapid response where hygiene, isolation, testing, and vaccination play the main roles. However, multiple factors are involved, not the least the indirect impact of the ongoing climatic change with changing ecological niche determinants and the possibility of counterintuitive ways of infection, e.g. by picking it up from a dog or cat. These questions highlight the need for an approach optimizing existing strategies with an eye on the possibility of future unexpected crises, a situation that demands developing and implementing an integral (e.g. joint surveillance of SARS-CoV-2 in humans and animal, and cross-sectoral collaboration), evidence-based referral network (e.g. transmission models, practical control options, and added value of scientific insights) to navigate the continuously changing COVID-19 situation.

Although COVID-19 is a human pandemic, various animal species are also at risk for infection and epidemic transmission due to the zoonotic character of SARS-CoV-2, with the extended possibility of re-transmission from animals to humans. According to FAO qualitative exposure assessment, the likelihood of exposure of humans or animals to SARS-CoV-2 in COVID-19 affected areas through contact with wild animals, company animals, livestock animals, and aquatic animals is different [8]. This is not as far-fetched as one might first think since hundreds of animal COVID-19 infections have currently been reported to the World Organization for Animal Health (OIE) [9]. In fact, a surveillance and response approach, including an early warning system (EWS), of SARS-CoV-2 infections in animals must urgently be contemplated.

The advantage of the One Health approach with respect to COVID-19 is not only reflected by closely linked and inter-dependent exchange of activities, but also influenced by the environmental variables that shape the various ecosystems we all live in. Given the broad dissemination in various environments and the fact that the virus can infect both domestic and wild animals [10], we propose applying a perspective considering animals and the environment in addition to the current human-centred COVID-19 strategy.

## Materials and methods

As we realize the transmission mode and route of COVID-19, mounting evidence-based strategies must be implemented to reduce COVID-19 infection rates, not only on humans but also with respect to animals.

### Zoonotic determinants

To encourage more attention to potential animal SARS-CoV-2 infections, it would be important to analyse the epidemiological characteristics of this virus occurred in those areas, including identification of the epidemic status in susceptible species and assessment of current animal-oriented control activities. To that end, we conducted a systematic database search using the following keywords: “COVID-19” and “SARS-CoV-2” together with “animal” in PubMed, Web of Science (WoS), and Google Scholar from the first outbreak in 2019 up to October 2021. We also used the Google search engine with search terms associated with our defined topics to identify potential additional information. Data were collated with respect to animal species, infection rates and outcomes mentioned in retrieved news and reports through multiple databases (including WHO and OIE). Additionally, a similar search strategy was applied for the topics of “Household and other enclosed spaces,” “Nosocomial infections,” “Transportation,” and “The market place” and “Role of the environment.” Criteria for eligibility were studies related to the COVID-19, and exclusion criteria were review articles, commentaries, letters, editorial, opinion, duplicated publications with previous studies, news, and studies without valid data.

## Results

Since 2019, more than 500 animal SARS-CoV-2 infection events were found to have been reported in 24 countries, including China, India, the UK, the USA, Latvia, and Switzerland. Indeed, the infection spans Asia, Africa, the Americas, and Europe and has even been found in Antarctica. The majority of these outbreaks occurred in central US (93.4%). With the elongation of the current SARS-CoV-2 pandemic, the risk of animal exposure is elevated, posing a threat to both human and animal health.

**Figure 2.**
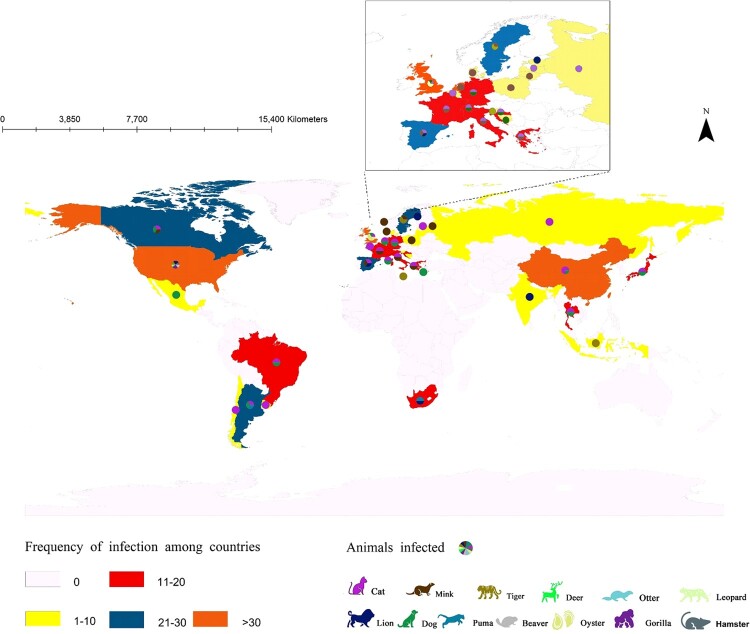
Reported COVID-19 prevalence in animals worldwide.

As the overall number of animals infected by SARS-CoV-2 increases, the number of infected species that have been identified also expands. So far, based on the data of infections collected, cats (23.3%) and dogs (22.5%) account for the highest proportion and have been reported in 16 countries. Tigers (14.7%) and leopards (14.0%) rank second, followed by a lower level of infections in gorillas (4.7%), lions (3.9%), and deer (3.1%) (Figure 2). Because of the close relationship between humans and pets, such as dogs and cats, the number of infections in the latter two species may increase even faster along with the current wave of human COVID-19 infections and become common in the future. It has been confirmed that infected humans often are the source of animal SARS-CoV-2 infections [11–13]. Although bat species are the perceived reservoir of SARS-CoV-2, animal-to-human transmission has not yet been identified beyond doubt, except for mink and hamster [14,15].

### Animal welfare in the context of COVID-19

Thousands of unattended pets, even livestock, have died or are at risk of dying after residents have been evacuated or considered abandoning or killing pets for fear of zoonotic transmission, with only very few infected animals receiving treatment [16]. After SARS-CoV-2 had been reported in farmed mink, many millions of minks were culled worldwide, particularly in Denmark, the Netherland, and Spain [17–19]. In addition, COVID-19 directly or indirectly disrupts activities to keep livestock healthy, including the prevention and control of animal diseases, such as veterinary diagnostics, disease surveillance, etc. [20].

Understanding the risk of people spreading SARS-CoV-2 to animals and vice versa is essential to the success of approaches implemented to control the current crisis. Based on the available evidence to date, animals have generally not been considered when discussing contracting or spreading COVID-19 to people. More evidence-based studies are needed to understand the impact of COVID-19 on animal management and welfare and if and how different animals (pets, zoo animals, farm animals, wild animals, and exotic animals) can be affected by COVID-19.

So far, the current outbreaks of animal infections have occurred mainly in homes (53.3%) and zoos (37.6%). Animal deaths due to COVID-19 have been reported in China, India, Latvia, Sweden, and the US, with events concentrated in cats (18.2%) and dogs (27.3%) and only few cases reported for wild animals [21,22]. Genome variants of SARS-CoV-2 have also been detected in marine organisms (shellfish clams, etc.) [23]. Excrements of patients (both human and animal) left in nature may result in transmission to animals and indeed aquatic organisms via contaminated flows of water, especially freshwater organisms [24,25]. In this way, infected animals can reduce safe drinkable freshwater resources and increase the risk of infection in the wild; this goes also for dead animals and carcasses left in nature [26–28].

### Household and other enclosed spaces

The exhaled fine droplets and aerosol particles from infected persons can build up in a confined space, attach to surfaces, and would be transferred through airflow or contact [29,30].

The household space represents the main place for SARS-CoV-2 transmission even though they generally only hold low numbers of people. COVID-19 spreads unhindered in households as they embody densely populated settings where basic recommendation to avoid infection cannot be upheld [31,32]. Both retrospective and prospective studies indicate that household transmission of SARS-CoV-2 occurs quickly and the first index patients confirmed and originated from either children or adults [33–35]. Thus, although households are limited in numbers, they are hotspots and act as a primary driving force for transmission, where the next step takes place when neighbours meet. In addition, the limited spaces shared, delays isolation of index patients, unvaccinated individuals, and even vaccinated family members with breakthrough infections thus facilitate the further spread of viral infection.

Outside the home, examples of other close-contact settings with inadequate ventilation include restaurants, bars, clubs, and particularly elevators, which are common in cities. Areas if this kind represents an additional risk due to the constant mix of people. In addition, extensive viral contamination has been detected from various surfaces. Studies have shown that the survival time of SARS-CoV-2 is related to the contact material. SARS-CoV-2 can survive in aerosols for about 3 h, up to 4 h on copper, and around 24 h on paper [36]. Active virus can last on plastic and stainless-steel surfaces for 2–3 days [37]. Furthermore, several studies have detected the genetic genome of SARS-CoV-2 in the human faeces of patients and related waste-water samples [38,39] and put forward the hypothesis of the environment transmission chain. However, there is no conclusive evidence of potential routes to ensure viral transmission to humans.

### Nosocomial infections

Improper prevention and control endanger patients and healthcare workers in hospitals and SARS-CoV-2 spill-over into outdoor hospital environments have worsened the pandemic [40,41]. At present, we have identified 24 reports of nosocomial infections involving multiple departments and wards in 11 countries, including China, Japan, the UK, the USA, Spain, etc.[42–48]. In Osaka, Japan, three hospitals that never admitted infected patients, still found cluster infections, which may have been caused by virus contamination or defects in the hospital prevention and control system [43]. The private hospital in KwaZulu Natal Province in South Africa has strict existing prevention and control programmes, and serious nosocomial infections are still occurring, suggesting that intensive and pervasive infection control procedures need to be improved [49].

### Transportation

Individual mobility within their social networks is the root cause for the spread of COVID-19. After the outbreak of the pandemic, countries around the world have adopted different levels of traffic restriction measures. Currently, imported COVID-19 cases have been reported by many countries, and international exportation is still the main channel for the imported COVID-19 cases. At the same time, imperfect prevention and control systems put countries having reached the control stage in situations where they fall back to full dissemination of the virus. Although most countries require a negative viral test within the expiration date for passengers in airplanes, trains, and cars in international traffic, imported COVID-19 play an important role in dissemination, e.g. infected flight passengers and staff resulting in community spill-over infections. Other types of transportation have also been associated with infections, such as buses and cruises. Covid-19 infected crew members in French military aircraft carrier and US military submarine have been reported, and the source of the infection is still under investigation [50].

A method of visualizing the spatial and chronological aspects of the spread of this virus based on geographical information systems (GIS) was recently reported [51], and a case study in Bangladesh found COVID-19 to be strongly associated with the reach and mechanism of transport networks [52]. Gephi graphs based on qualitative data from newspaper reports and prepared layouts varying from macro to micro scales show that this approach can enrich traditional GIS approaches, thereby assisting mobility planners and policymakers [53].

### The market place

SARS-CoV-2-infested infections from the grocery store, supermarket, and wholesale market have links with the outbreak and re-emerging outbreak of COVID-19 in China [54] and the USA [55]. Although it is generally safe if existing protections and social distancing guidelines are followed, workers from industries face some of the highest risks of COVID exposure to infected customers or cold-chain contamination in the frozen food or its packaging [56–59]. Despite uncertainties in the potential transmission mechanism, the live SARS-CoV-2 virus was isolated from food products, packaging, or cargo containers in the cold chain. SARS-CoV-2 transmission in the food supply chain during the COVID-19 pandemic should be on high alert, and enhanced surveillance on food supply, fast SARS-CoV-2 detection, secured vaccinations for frontline workers is warranted.

### Role of the environment

**Figure 3.**
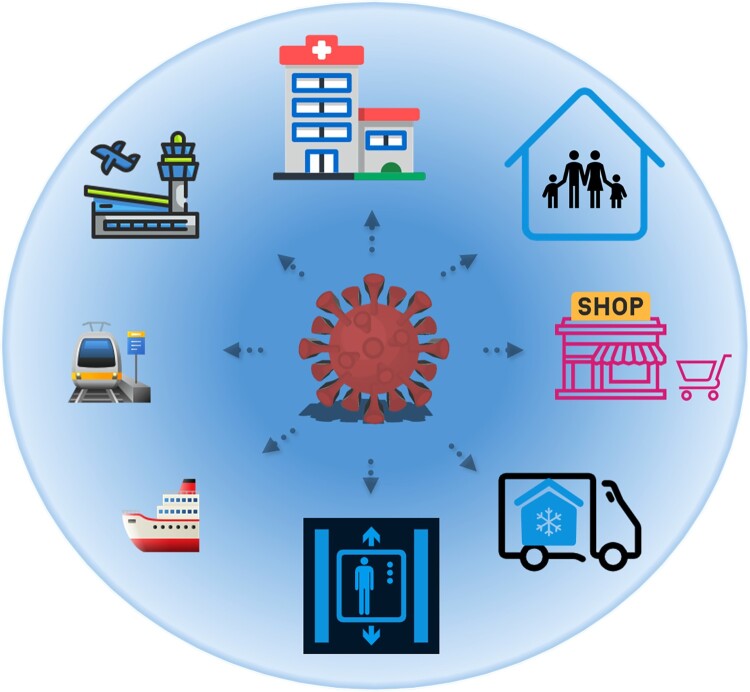
Common types of environments that posing high transmission risk.

Although COVID-19 can be introduced between living organisms, it should be remembered that it can also take place when handling different items as the virus can survive long time on most surfaces [60–62]. The built environment pays an important role in bringing many persons together in closed areas, such as households, hospitals, airports, supermarkets, and other public places, as well as elevators, bars, and other airtight closed environments (Figure 3). Through literature and news searches, we analysed actual environmental that can cause transmission. At present, we have collected reports of cluster infections in 28 high-risk environments in Canada, China, Japan, South Korea, and the USA. Most environmental infection-related incidents occur in crowded cities in economically developed cities. However, it remains unclear about SARS-CoV-2 spill-over effect of environmental fomites, and the role of the environment could play in pathogenesis, spread, and severity of COVID-19 [30]. In addition, the COVID-19 has brought both positive and negative environmental impacts, especially warning and response from nature would alert humans and provide insights for contamination on further aggravation of the pandemic [63].

## Discussion

Although the exact origin of SARS-CoV-2 has not yet been determined, all existing data and speculations point to wild animals as reservoirs. When the first human COVID-19 cases were diagnosed by the end of 2019, the China National People's Congress passed legislation prohibiting the consumption of any meat from killed or kept captive, wild animals to protect the health of the public. However, current wildlife protection law does not have a clear definition of wildlife, and some animals under national protection are allowed to be bred commercially, such as the plum deer. In addition, the coverage of wildlife species is incomplete and has not been updated since nearly 30 years. Such loopholes provide opportunities for illegal wildlife trade, which contributes to increasing the risk of emerging infectious diseases.

Animals and humans are often in close contact, e.g. cross-infection is possible when dealing with domestic animals and when it comes to pets man and animal are close to inseparable. The current pandemic has diminished the importance of animal welfare and thrown up unparalleled challenges for animal management. In the early stages of the pandemic, the primary focus of the policy was on healthcare in humans, and it still predominantly is. However, unexpectedly, isolation or quarantine to contain the spread of COVID-19 constituted a horrific threat to animal welfare. The main approach for animal management still depends on animal shelters, quarantine with owners, automatic feeders, etc. There is a need to address animal welfare with greater attention and better awareness.

Hospitals cannot avoid gathering infections within closed environments, and COVID-19 infections associated with healthcare are common, in particular with regard to doctors and nurses. Possible links to transmission risk are an ongoing topic of research.

However, whether the disease is caused by viruses, bacteria, parasites, or fungi, the control process is not focused only on humans. The human-animal-human SARS-CoV-2 transmission has been demonstrated, together with the presence and persistence of SARS-CoV-2 in waste-water, which made it a potential to become an environmental reservoir and route for SARS-CoV-2 transmission remain unanswered.

Usually, environment means the physical surroundings existing in some kind of relation to human-centred, including both material factors such as air, water, land, plants, and animals. A more generalized concept of environment contains non-material parts such as ideas, institutions, and behavioural norms [64]. The increased use and disposal of disinfectants, masks, and gloves for SARS-CoV-2 control continuously endanger the environment. An emerging group of studiesis showing that climate change plays an important role in the global transmission of SARS-CoV-2 [65]. Exposure to air pollution may influence COVID-19 transmission by increasing the susceptibility to infection and mortality [66,67]. The disturbance of ecological balance due to forest degradation has also been linked to the spread of COVID-19 [68,69]. The COVID-19 pandemic has led to large-scale behaviour change reflected not only in isolated social distancing but also by changing psychological conditions and core values [70].

The emergence of SARS-CoV-2 is a complex process, with a wide range of hosts, environments, and various factors enabling the virus to mutate and evolve. Large-scale intensive agriculture, aquaculture, and animal markets, human encroachment on wildlife habitats due to agricultural, industrial, and urbanization needs, and even the development of global tourism and climate change all exert an impact on the interaction between humans, animals, and the environment. In response to these issues, One Health can provide a helpful approach when formulating policies to promote the prevention and control of emerging infectious diseases like the COVID-19 pandemic beyond a human-centred framework. In addition to risk surveillance in humans, strong vigilance should be paid to the flow and spill-over risk of the virus from animals or the environment to humans through genetic and epidemiological surveys to avoid onward human-to-human transmission.

## Conclusion

The importance of the One Health approach is in its recognition of the closely linked and inter-dependent mutual effect between humans, animals, the environment, and broader ecosystems. Given that the virus has infected companion and wild animals, together with extensive environmental contamination detected, we propose the consideration of animals and the environment beyond the human-centred COVID-19 prevention and control strategy under One Health perspective, and also highlight the value of promoting one health activity to improve coordination across global preparedness to the pandemic in the future.
